# 3′ Splice Site Sequences of Spinal Muscular Atrophy Related SMN2 Pre-mRNA Include Enhancers for Nearby Exons

**DOI:** 10.1155/2014/617842

**Published:** 2014-01-27

**Authors:** Sunghee Cho, Heegyum Moon, Tiing Jen Loh, Hyun Kyung Oh, Hey-Ran Kim, Myung-Geun Shin, D. Joshua Liao, Jianhua Zhou, Xuexiu Zheng, Haihong Shen

**Affiliations:** ^1^School of Life Sciences, Gwangju Institute of Science and Technology, Gwangju 500-712, Republic of Korea; ^2^Department of Laboratory Medicine, Chonnam National University Medical School and Chonnam National University Hwasun Hospital, Hwasun 519-763, Republic of Korea; ^3^The Hormel Institute, University of Minnesota, Austin, MN 55912, USA; ^4^Jiangsu Key Laboratory of Neuroregeneration, Nantong University, Nantong, China

## Abstract

Spinal muscular atrophy (SMA) is a human genetic disease which occurs because of the deletion or mutation of SMN1 gene. SMN1 gene encodes the SMN protein which plays a key role in spliceosome assembly. Although human patients contain SMN2, a duplicate of SMN1, splicing of SMN2 produces predominantly exon 7 skipped isoform. In order to understand the functions of splice site sequences on exon 7 and 8, we analyzed the effects of conserved splice site sequences on exon 7 skipping of SMN2 and SMN1 pre-mRNA. We show here that conserved 5′ splice site sequence of exon 7 promoted splicing of nearby exons and subsequently reduced splicing of distant exons. However, to our surprise, conserved 3′ splice site sequence of exon 7 and 8 did not promote splicing of nearby exons. By contrast, the mutation inhibited splicing of nearby exons and subsequently promoted splicing of distant exons. Our study shows that 3′ splice sites of exon 7 and 8 contain enhancer for their splice site selection, in addition to providing cleavage sites.

## 1. Introduction

Pre-mRNA splicing is a process that removes introns and ligates exons. Pre-mRNA splicing is required for the gene expression in higher eukaryotes [1, 2]. Pre-mRNA splicing occurs in two consecutive steps. In the first step, 5′ splice site is cleaved by the attack of branch-point Adenine residue, and 2′–5′ phosphodiester bond is formed to produce a lariat structure [1]. In the second step, the 3′ splice site is cleaved and two exons are ligated. The sequence of splice sites in human is heterogenous, and thereby switching the splice sites into the conserved sequence promotes splicing reaction.

In the alternative splicing, different exons are included in different mature RNAs to produce various proteins from a single gene [3–5]. The inclusion or skipping of exons is regulated by cis-acting or trans-acting elements. Cis-acting elements are the RNA sequence on pre-mRNA which promotes or inhibits exon inclusion or skipping. Trans-acting elements are the proteins that regulate alternative splicing. The best understood trans-acting elements are SR proteins and hnRNPs [6–10].

Spinal muscular atrophy (SMA) is an autosomal recessive disorder in which motor neurons of spinal cord degenerate [11, 12]. SMA occurs because SMN1 gene is deleted or mutated [13]. SMN1 gene encodes SMN protein which has an important function in spliceosome assembly [14–16] and the transport of *β*-actin in neurons [17–21]. However, all of human patients have SMN2 gene, a duplicate of SMN1 gene, in which there are 5-point mutations compared with SMN1 gene [13, 22]. The major difference of SMN1 and SMN2 pre-mRNA splicing is the alternative splicing exon 7 [23, 24]. SMN1 pre-mRNA produces exon 7 included form predominantly which encodes the functional SMN protein [13]. SMN2 pre-mRNA produces exon 7 skipped form predominantly which does not encode the functional SMN protein [25, 26]. It is striking that a C to T point mutation at exon 7 of SMN2 induces such a significant decrease of exon 7 inclusion.

It has been shown that exon 7 of SMN2 contains enhancer and inhibitor of exon 7 inclusion [27–30]. The C to T mutation on SMN2 produces the binding sites for hnRNP A1 to inhibit exon 7 inclusion [31]. Tra2*β* binds to exon 7 to promote exon 7 inclusion [32, 33]. In addition to exon 7, intron 6 and intron 7 also contain regulatory elements [34–38].

In order to understand the function of splice sites on exons 6–8, we mutated the splices sites to conserved sequences. We show here that conserved 5′ splice site of exon 7 promotes the splicing of nearby exons. However, surprisingly, conserved 3′ splice sites of exon 7 and exon 8 did not promote its splicing but inhibited splicing. Our results indicate that the 3′ splice sites of exon 7 and exon 8 include enhancer sequence of exon 7 inclusion of SMN2 pre-mRNA.

## 2. Materials and Methods

### 2.1. Plasmid Construction

All primers used for constructs are listed in Table 1. The SMN1 and SMN2 sequences were PCR-amplified using human genomic DNA as a template to construct wild type SMN1 and SMN2 minigenes. To generate SMN1-S and SMN2-S minigenes, we performed overlapping PCR reactions to delete part of intron 6. In the first PCR reaction, SMN1-GFP and SMN2-GFP [39, 40] constructs were used as templates, and SMNE6(B)F and SMNI6(D)R were used as forward and reverse primers. In the second PCR, SMNI6(D)F and SMNE8(X)R primers were used as forward and reverse primers, and the genomic DNA (Promega) was used as the template. We cloned the third PCR products into pcDNA3.1(+) plasmid using restriction enzymes BamHI and XhoI. To generate SMN1(2)-E7-5′ss-con, and SMN1(2)-E7-3′ss-con, SMN1(2)-E8-3′ss-con mutants, we performed overlapping PCR. The following primers were used to perform the site-directed mutagenesis; common primers (forward: SMNE6(B)F, reverse: SMNE8(X)R), specific primers for SMN1-E7-5′ss-con and SMN2-E7-5′ss-con (forward: E75′CSF, reverse: E75′CSR), SMN1-E7-3′ss-con and SMN2-E7-3′ss-con (forward: E73′CSF, reverse: E73′CSR), and SMN1-E8-3′ss-con and SMN2-E8-3′ss-con (forward: E83′CSF, reverse: E83′CSR).

### 2.2. RT-PCR

RiboEx reagent (GeneAll) was used to extract total RNA transfected cells. 1 *μ*g of RNA sample was used per 2 *μ*L reaction with oligo (dT) primer and ImProm-IITM reverse transcriptase (Promega). 1 *μ*L of cDNA was amplified by PCR with G-Taq polymerase (Cosmo Genetech). The primers for SMN1-S, SMN2-S minigene, and conserved splice site constructs are as follows: forward: SMN.Fwd (5′-ATA ATT CCC CCA CCA CCT CC-3′), reverse: pCDNA.Rev (5′-CTA GAA GGC ACA GTC GAG GCT-3′) which specifically anneal to the plasmid backbone. The GAPDH mRNA was amplified with primers GAPDHFwd (5′-ACC ACA GTC CAT GCC ATC A-3′) and GAPDHRev (5′-TCC ACC ACC CTG TTG CTG TA-3′). Splicing products were amplified using 20 PCR cycles (94°C for 30 s, 60°C for 30s, and 72°C for 30 s). The PCR products from SMN minigene constructs were loaded onto 2% agarose gels. These gels could be visualized by staining with ethidium bromide solution (0.5 *μ*g/mL). Intensity of the approximate bands was quantified using the ImageJ Software (National Institutes of Health, Bethesda, MD).

### 2.3. Cell Culture and Transfection

C33A and 293A cells were grown in Dulbecco's Modified Eagle's Medium (DMEM) supplemented with 10% of fetal bovine serum (FBS) and antibiotics (100 U/mL penicillin G and 100 *μ*g/mL streptomycin) at 37°C. All cells were maintained in a humidified 5% CO_2_ condition. We used polyethyleneimide (PEI) reagent to transfect plasmids into cells. 1 *μ*g of PEI was mixed with 0.5 *μ*g of plasmid in 100 *μ*L of DMEM, then the mixture was applied to cells in 900 *μ*L of DMEM supplemented with 10% FBS. Four hours later, we changed the supernatant with fresh medium. The cells were incubated for 48 hours before extraction of RNA.

## 3. Results

### 3.1. Splicing of SMN1 and SMN2 Minigenes Recapitulates Endogenous Splicing in C33A Cell Line

**Figure 1:**
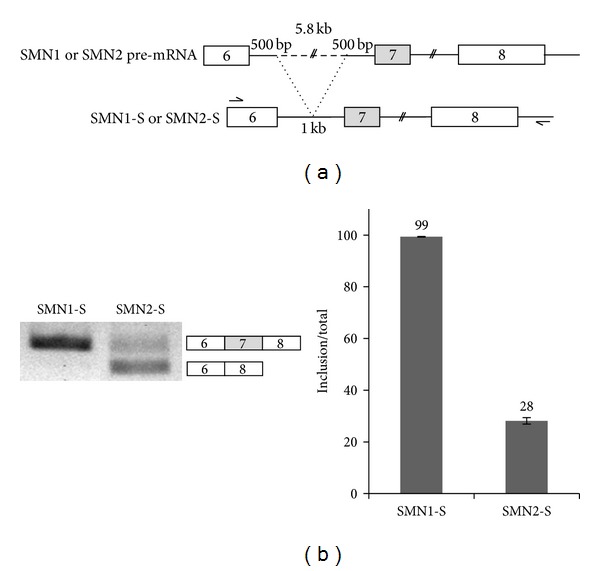
Splicing of SMN1 and SMN2 minigenes recapitulates endogenous splicing in C33A cell line. (a) Minigene constructs of SMN1-S and SMN2-S. The 4719 nt of intron 6 is deleted from both constructs. Primers used in RT-PCR are also shown. (b) RT-PCR analysis of SMN1 and SMN2 minigenes. GAPDH was used as the control. Quantitation results are shown.

To determine the effects of splice sites on exon 7 splicing of SMN2 and SMN1 genes, we constructed minigenes which contain exons 6–8 of SMN1 and SMN2 genomic DNA. As shown in Figure 1(a), in the minigene constructs, the ~5.8 kb of intron 6 was deleted, but flanking 0.5kb DNA remained intact, named as SMN1-S and SMN2-S. After the SMN2-S and SMN1-S minigenes were transfected into C33A cells, RNA was extracted for RT-PCR. Consistent with endogenous splicing of SMN1, as shown in Figure 1(b), exon 7 included isoform was detected predominantly (~90%) in the SMN1-S minigene, whereas exon 7 skipped form was hardly detectable. However the splicing of SMN2-S minigene showed significant level of exon 7 skipping (~70%) (Figure 1(b)). Because the SMN1-S and SMN2-S minigenes recapitulate the endogenous exon 7 splicing, we decided to use them to identify the cis-acting elements on splice sites of SMN2 pre-mRNA.

### 3.2. Conserved Sequence on 5′ Splice Site of Exon 7 Promotes Exon 7 Inclusion on SMN2 Pre-mRNA

**Figure 2:**
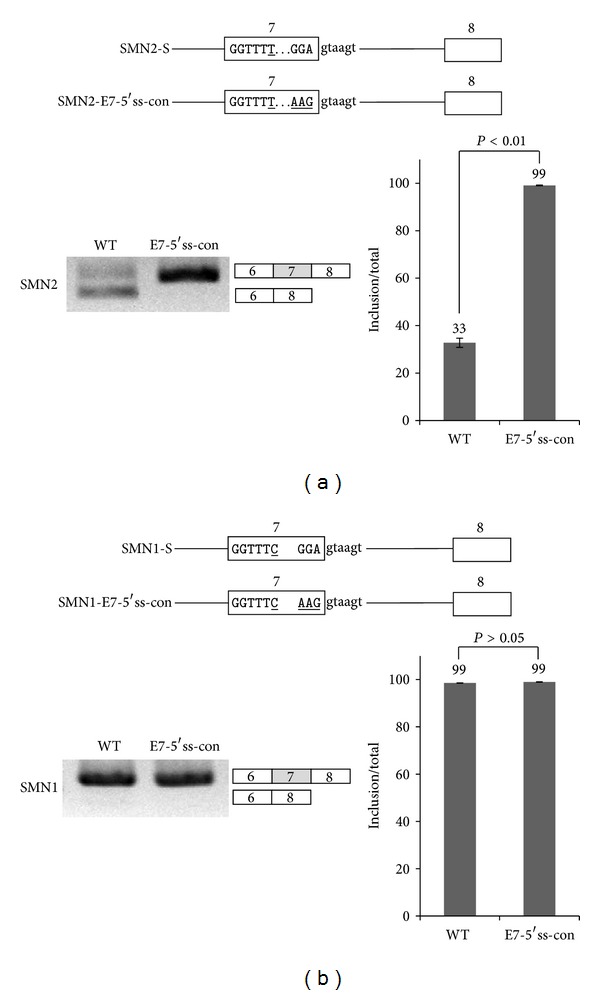
Conserved sequence on 5′ splice site of exon 7 promotes exon 7 inclusion on SMN2 pre-mRNA. (a) A mutant construct (SMN2-E7-5′ss-con) in which 5′ splice site of exon 7 was mutated to the conserved sequence in SMN2 is shown. RT-PCR analysis of SMN2-E7-5′ss-con minigene of SMN2 pre-mRNA. (b) A mutant construct (E7-5′ss-con) in which 5′ splice site of exon 7 was mutated to the conserved sequence in SMN1 is shown. RT-PCR analysis of E7-5′ss-con minigene of SMN1 pre-mRNA is shown.

In order to ask if the splice sites sequences in the exon 7 and 8 of SMN2 pre-mRNA include cis-acting elements for exon 7 skipping/inclusion of SMN2 pre-mRNA, we performed mutagenesis assay on the splice sites on SMN2-S minigene. In the first approach, we mutated 5′ splice site of SMN2 exon 7 from GGAgtaagt to a conserved sequence-AAGgtaagt (SMN2-E7-5′ss-con) (Figure 2(a)). We predict that the SMN2-E6-5′ss-con mutant, which contains conserved 5′ splice site sequence at exon 7, provides stronger splicing activity at exon 7, and thus the strength of 5′ splice site of exon 6 becomes relatively weak. Subsequently, exon 7 inclusion will be promoted on the E7-5′ss-con mutant. Consistent with our prediction, as shown in Figure 2(a), exon 7 inclusion is significantly increased (~99%). Therefore we conclude that the conserved sequence at 5′ splice site of exon 7 promotes exon 7 inclusion on SMN2 pre-mRNA. We next asked if the conserved sequence at 5′ splice site of exon 7 also promotes exon 7 skipping of SMN1-S minigene pre-mRNA. We performed similar mutagenesis assay on the SMN1 minigene to mutate 5′ splice site of exon 7 into a conserved sequence (SMN1-E7-5′ss-con) (Figure 2(b)). Similar to the SMN2-E7-5′ss-con minigene, we predict that the conserved sequence at 5′ splice site of exon 7 promotes exon 7 skipping. Because the exon 7 skipped isoform is not detected in the SMN1 minigene, we also predict that SMN1-E7-5′ss-con mutant of SMN1 produces exon 7 included isoform only. As we predicted, exon 7 included isoform was predominantly detected from the E7-5′ss-con mutant whereas exon 7 skipped isoform was hardly detected. To combine the results of Figures 2(a) and 2(b) together, we conclude that 5′ splice site of exon 7 does not include additional cis-acting elements for exon 7 splicing of SMN1 and SMN2 pre-mRNA.

### 3.3. 3′ Splice Site of Exon 7 Includes Enhancer for Exon 7 Inclusion of Both SMN2 and SMN1 Pre-mRNA

**Figure 3:**
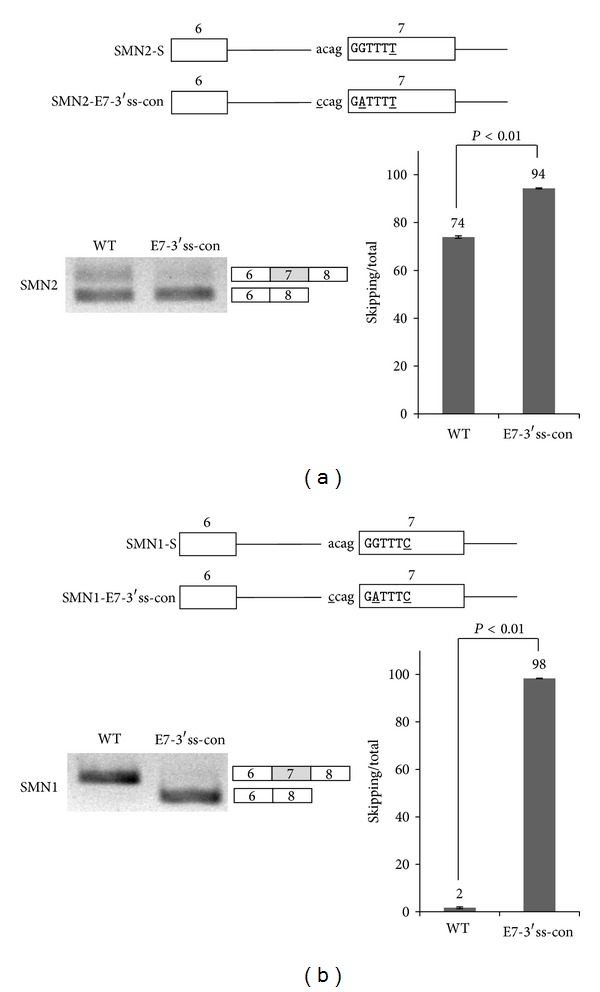
3′ splice site of exon 7 includes enhancer for exon 7 inclusion of both SMN2 and SMN1 pre-mRNA. (a) A mutant construct (SMN2-E7-3′ss-con) in which 3′ splice site of exon 7 was mutated to the conserved sequence in SMN2. RT-PCR analysis of SMN2-E7-3′ss-con minigene of SMN2 pre-mRNA is shown. (b) A mutant construct (SMN1-E7-3′ss-con) in which 5′ splice site of exon 7 was mutated to the conserved sequence in SMN1 is shown. RT-PCR analysis of E7-3′ss-con minigene of SMN1 pre-mRNA is shown.

As the second approach, we asked if the strong 3′ splice site on exon 7 affects exon 7 splicing. We noticed that the 3′ splice site on exon 7 is as strong as the conserved sequence. Therefore we hypothesized that the mutation of the 3′ splice site into a conserved sequence would not affect exon 7 splicing. We mutated the 3′ splice site sequence of SMN2 and SMN1 exon 7 from acagGG to a conserved sequence-ccagGA (SMN2-E7-3′ss-con and SMN1-E7-3′ss-con) (Figures 3(a) and 3(b)). We transfected the mutant constructs into 293A cells and then performed RT-PCR analysis with the extracted RNA. However, to our surprise, exon 7 skipping was significantly promoted on the SMN2-E7-3′ss-con mutant as compared with the wild type SMN2 minigene (from ~70% to ~94%) (Figure 3(a)). Thus we conclude that there is a cis-element on the 3′ splice site signal which enhances exon 7 inclusion and that our mutagenesis destructed the enhancer function. We next asked if the 3′ conserved sequence on exon 7 of SMN1 pre-mRNA has the similar effects on exon 7 splicing. The results in Figure 3(b) showed that the mutation of 3′ conserved sequence produced exon 7 skipped isoform predominantly on SMN1 exon 7 also promotes exon 7 skipping (~98%), whereas the wild type SMN1-S produced exon 7 included form predominantly. Thus the enhancer at 3′ splice site signal also functions at SMN1 pre-mRNA splicing. The results in Figures 3(a) and 3(b) indicate that the enhancer function is not dependent on the point mutation on exon 7 of SMN2 pre-mRNA. To combine the results in Figures 3(a) and 3(b), we conclude that 3′ splice sites of exon 7 include enhancer sequence for exon 7 inclusion for both SMN1 and SMN2 pre-mRNA.

### 3.4. 3′ Splice Site of Exon 8 Also Includes an Enhancer for Exon 7 Inclusion

**Figure 4:**
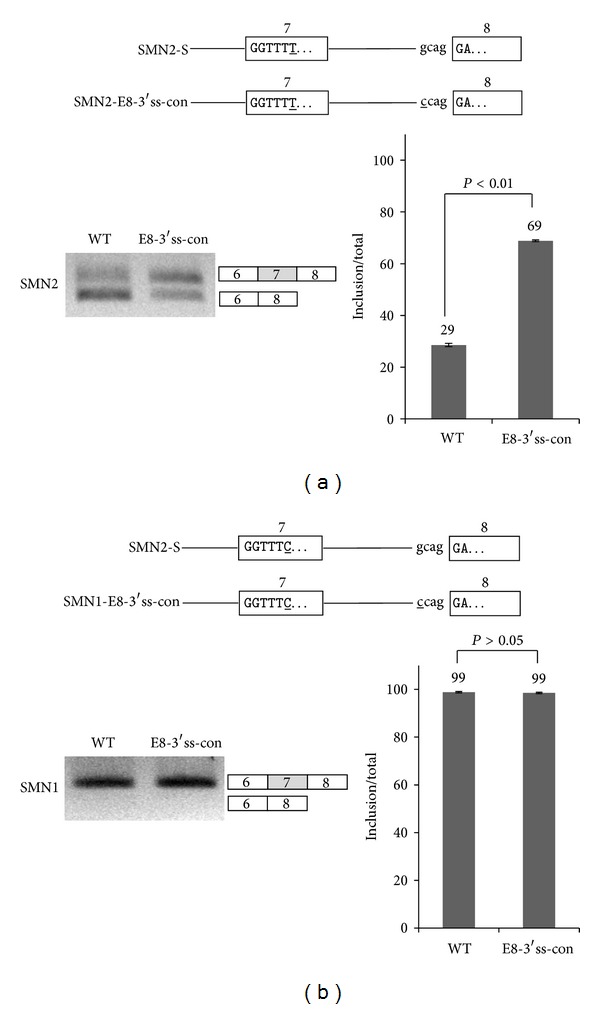
3′ splice site of exon 8 also includes an enhancer for exon 7 inclusion. (a) A mutant construct (E8-3′ss-con) in which 3′ splice site of exon 7 was mutated to the conserved sequence in SMN2. RT-PCR analysis of SMN2-E8-3′ss-con minigene of SMN2 pre-mRNA is shown. (b) A mutant construct (SMN1-E8-3′ss-con) in which 5′ splice site of exon 7 was mutated to the conserved sequence in SMN1. RT-PCR analysis of SMN1-E8-3′ss-con minigene of SMN1 pre-mRNA.

As the last approach, we asked if the stronger 3′ splice site on exon 8 also affects exon 7 splicing. We found that the 3′ splice site on exon 8 is as strong as the conserved sequence. Therefore we had a similar hypothesis that the mutation of the 3′ splice site into a conserved sequence would not affect exon 7 splicing. We mutated the 3′ splice site of exon 8 from gcagGA into a conserved sequence-ccagGA in the SMN2 and SMN1 minigenes (SMN2-E8-3′ss-con and SMN1-E8-3′ss-con) (Figures 4(a) and 4(b)). RT-PCR results of the mutants showed that, to our surprise, the change of 3′ splice site of exon 8 into a conserved sequence (E8-3′ss-con) promoted exon 7 inclusion significantly (~63%) (Figure 4(a)). The promotion of exon 7 inclusion indicates that the cleavage at 3′ splice site of exon 7 will become easier and that the cleavage at 3′ splice site of exon 8 will become harder. Thus the 3′ splice site signal at exon 8 also includes an enhancer, and our point mutagenesis disrupted its function as an enhancer. Therefore 3′ splice site of exon 8 includes an enhancer for exon 7 inclusion of SMN2 pre-mRNA. We next asked if the 3′ conserved splice sites of SMN1 exon 8 have the similar effects as those on SMN2 pre-mRNA. The results in Figure 4(b) showed that only exon 7 included isoform was detected for the SMN1-E8-3′ss-con mutant. Because SMN1-S minigene expressed exon 7 included isoform predominantly, the increase of exon 7 inclusion is hardly detectable. Thus we conclude that the conserved splice site at 3′ splice site of exon 8 had a similar effects on SMN1-E8-3′ss-con mutant (Figure 4(b)). The results in Figures 4(a) and 4(b) demonstrate that 3′ conserved splice sites of exon 8 contain an enhancer that promotes exon 8 inclusion and then subsequently inhibits exon 7 inclusion.

## 4. Discussion

In this study, we analyzed the effects of splice site sequences of SMN1 and SMN2 pre-mRNA on exon 7 splicing. We tested the prediction that if the splice site signal of one exon becomes stronger by mutating into conserved sequence, then the splicing signal of the other exon will become relatively weak. If the mutation to a conserved sequence does not have the predicted consequences, then we can conclude that the splice site includes enhancer or inhibitor sequences. The mutagenesis of 5′ splice site at exon 7 into the conserved sequences followed this prediction. Conserved sequence at 5′ splice site of exon 7 promotes exon 7 inclusion. The results indicate that the mutation to a conserved sequence promotes the usage of 5′ splice site on exon 7, and, consequently, the usage of 5′ splice site on exon 6 is reduced, and thereby exon 7 inclusion is promoted. In contrast to the effects of 5′ splice sites on splicing, conserved sequence at 3′ splice sites inhibited splicing of nearby exons. Mutation of the 3′ splice site sequence on exon 7 to a conserved sequence promoted exon 7 skipping of both SMN2 and SMN1; thus we conclude that the 3′ splice site contains an enhancer sequence that promotes exon 7 inclusion. The mutagenesis disrupted the enhancer function. Similarly, conserved sequence of 3′ splice site at exon 8 promoted exon 7 inclusion of both SMN2 and SMN1; thus we conclude that 3′ splice site on exon 8 includes an enhancer for exon 8 splicing, and consequently exon 7 splicing is inhibited. Therefore we conclude that 3′ splice sites at exon 7 and 8 contain splicing enhancer in addition to providing cleavage signals.

### 4.1. 5′ Splice Site of Exon 7 Functions Only as Splice Sites in SMN2 Pre-mRNA

We found that the mutation of 5′ splice sites into the conserved sequence promoted splicing of nearby exons and thus reduced splicing of distant exons. Our results indicate that 5′ splice sites on exon 7 of SMN2 pre-mRNA are conventional splice sites with less conserved sequences. Thus promoting exon 7 inclusion of SMN2 is possible by simply engineering the 5′ splice sites to conserved sequences.

### 4.2. 3′ Splice Site Sequences of Exon 7 and 8 Include Splicing Enhancers for Nearby Exons

Our results showed that the mutation of 3′ splice sites into the conserved sequence instead of promoting the splicing of nearby exons promotes the splicing of distant exons. Our results are striking because the 3′ splice sites cleavage did not follow the conventional splice site selection. The possible explanation is that 3′ splice sites contain enhancer sequence for splicing of nearby exons in addition to providing cleavage site. The mutation to the conserved sequence destructed the enhancer function for the splicing of nearby exons. Because a function of enhancer is to provide the binding sites for SR protein to promote splicing, we tried to predict the potential SR proteins which bind the RNA sequence with ESE finder but were not successful. The functional mechanisms of the enhancer need to be identified.

## 5. Conclusions

1. Splicing of SMN1 and SMN2 minigenes recapitulates endogenous splicing in C33A cell line.
2. Conserved sequence on 5′ splice site of exon 7 promotes exon 7 inclusion on SMN2 pre-mRNA.
3. 3′ splice site of exon 7 includes enhancer for exon 7 inclusion of both SMN2 and SMN1 pre-mRNA.
4. 3′ splice site of exon 8 also includes an enhancer for exon 7 inclusion.

## Figures and Tables

**Table 1:** Primer lists.

Name	Sequence
SMNE6(B)F	5′-TAC TCG GAT CCA TAA TTC CCC CAC CAC CTC C-3′
SMNI6(D)R	5′-GAC CTC TAA TCC CAG CTA CGA CAG GCG TGG TGG CAG-3′
SMNI6(D)F	5′-CTG CCA CCA CGC CTG TC G TAG CTG GGA TTA GAG GTC-3′
SMNE8(X)R	5′-CTA ACC TCG AGA ACA GTA CAA TGA ACA GCC ATG-3′
E75′CSR	5′-TGC CAG CAT TTC CTG GAA ATG AGA AA-3′
E75′CSF	5′-TTT CTC ATT TCC AGG AAA TGC TGG CA-3′
E73′CSR	5′-TTG TCT AAA ATC CTG GAA GGA AAA TA-3′
E73′CSF	5′-TAT TTT CCT TCC AGG ATT TTA GAC AA-3′
E83′CSR	5′-TGC CAG CAT TTC CTG GAA ATG AGA AA-3′
E83′CSF	5′-TTT CTC ATT TCC AGG AAA TGC TGG CA-3′
